# Preoperative Dose-Escalated Intensity-Modulated Radiotherapy (IMRT) and Intraoperative Radiation Therapy (IORT) in Patients with Retroperitoneal Soft-Tissue Sarcoma: Final Results of a Clinical Phase I/II Trial

**DOI:** 10.3390/cancers15102747

**Published:** 2023-05-13

**Authors:** Katharina Seidensaal, Matthias Dostal, Andreas Kudak, Cornelia Jaekel, Eva Meixner, Jakob Liermann, Fabian Weykamp, Philipp Hoegen, Gunhild Mechtersheimer, Franziska Willis, Martin Schneider, Jürgen Debus

**Affiliations:** 1 Department of Radiation Oncology, Heidelberg University Hospital, 69120 Heidelberg, Germany; 2 Heidelberg Institute of Radiation Oncology (HIRO), 69120 Heidelberg, Germany; 3 National Center for Tumor Diseases (NCT), 69120 Heidelberg, Germany; 4 Heidelberg Ion-Beam Therapy Center (HIT), Department of Radiation Oncology, Heidelberg University Hospital, 69120 Heidelberg, Germany; 5 Institute of Pathology, University of Heidelberg, 69120 Heidelberg, Germany; 6 Department of General, Visceral and Transplantation Surgery, University Hospital Heidelberg, 69120 Heidelberg, Germany; 7 Clinical Cooperation Unit Radiation Oncology, German Cancer Research Center (DKFZ), 69120 Heidelberg, Germany; 8 German Cancer Consortium (DKTK), Partner Site Heidelberg, 69120 Heidelberg, Germany

**Keywords:** retroperitoneal sarcoma, radiotherapy, IORT, intraoperative radiotherapy, dose-escalated radiotherapy, simultaneous integrated boost

## Abstract

**Simple Summary:**

Retroperitoneal sarcomas represent a very rare entity. The most common pattern of recurrence and cause of death is local recurrence, and the rates of locoregional recurrences are high even at high-volume centers. In contrast to soft-tissue sarcomas of the extremities, the role of radiotherapy in retroperitoneal sarcoma is not fully established. The aim of the study was to report the results of a prospective single-center trial for preoperative dose-escalated intensity-modulated radiotherapy with an intraoperative boost in patients with retroperitoneal sarcoma after all surviving patients had achieved a follow-up of at least 60 months. The primary endpoint of a 5-year local control of 70% was not met; the local control of the cohort was 59.6%. In those patients who received a dose > 50 Gy and the intraoperative boost, the local control was promising at 64.8%.

**Abstract:**

Background: To report the final results of a prospective, one-armed, single-center phase I/II trial (NCT01566123). Methods: Between 2007 and 2017, 37 patients with primary or recurrent (N = 6) retroperitoneal sarcomas were enrolled. Treatment included preoperative IMRT of 45–50 Gy with a simultaneous integrated boost of 50–56 Gy, surgery and IORT. The primary endpoint was local control (LC) at 5 years. The most common histology was dedifferentiated liposarcoma (51%), followed by leiomyosarcoma (24%) and well-differentiated liposarcoma (14%). The majority of lesions were high-grade (FNCLCC G1: 30%, G2: 38%, G3: 27%, two missing). Five patients were excluded from LC analysis per protocol. Results: The minimum follow-up of the survivors was 62 months (median: 109; maximum 162). IORT was performed for 27 patients. Thirty-five patients underwent gross total resection; the pathological resection margin was mostly R+ (80%) and, less often, R0 (20%). We observed 10 local recurrences. The 5-year LC of the whole cohort was 59.6%. Eleven patients received a dose > 50 Gy plus IORT boost; LC was 64.8%; the difference, however, was not significant (*p* = 0.588). Of 37 patients, 15 were alive and 22 deceased at the time of final analysis. The 5-year OS was 59.5% (68.8% per protocol). Conclusions: The primary endpoint of a 5-year LC of 70% was not met. This might be explained by the inclusion of recurrent disease and the high rate of G3 lesions and leiomyosarcoma, which have been shown to profit less from radiotherapy. Stratification by grading and histology should be considered for future studies.

## 1. Introduction

Retroperitoneal sarcomas (RPSs) constitute 15% of all soft-tissue sarcomas [1]. High-grade tumors are the most common; the spectrum of histological diagnoses is broad and ranges from lipo- and leiomyosarcoma to less common diagnoses [2,3]. In contrast to extremity soft-tissue sarcomas, local control (LC) is the central issue in the treatment of retroperitoneal sarcomas. Local recurrences are quite common and represent the leading cause of death. RPSs remain asymptomatic without specific symptoms for a long time; thus, many patients are diagnosed with large tumors of 16–21 cm median tumor size [2]. The primary treatment for initial and recurrent disease is surgery; however, incomplete resection with microscopic positive margins occurs in up to 65% of cases due to the immense size these tumors commonly achieve and the complex anatomy of the retroperitoneum [4]. Compartmental resection of organs adjacent to the tumor is the current surgical technique and has increased LC [5]. Although many undergo several consecutive multivisceral resections, the outcomes of retroperitoneal sarcomas are substantially less satisfactory compared to soft-tissue sarcomas at other sites. As known from extremity soft-tissue sarcoma, preoperative radiotherapy has the potential to increase LC; however, the available data are still limited, and further insight is needed. The only prospective trial published so far is the EORTC STRASS trial. The results did not support a broad use of radiotherapy in RPS and were contradictory to many other publications [1]. In an additional analysis, the results from STRASS have been pooled in a propensity-score-matched analysis with patients treated outside the trial (STREXIT). A benefit of additional radiotherapy was shown especially for patients with well-differentiated liposarcoma (WDLS) and dedifferentiated liposarcoma G1 and G2 [6,7]. The anatomy of the retroperitoneum complicates not only surgery but also radiotherapy; clinical target volume (CTV) margins known from extremity soft-tissue sarcomas cannot be adopted due to the necessary limitations to the adjacent organs at risk, mainly the bowel. Previous analyses have demonstrated that recurrence commonly occurs at the posterior margin of the tumor. Therefore, a simultaneous integrated boost (SIB) was tested on this high-risk margin with photon IMRT and protons before, with promising results, although with only a comparably short follow-up [8]. Intraoperative radiation therapy (IORT) has additional potential to increase the dose to the high-risk margin as identified during the resection. Herein, we present the final results of a phase I/II feasibility trial which combined a photon IMRT with a SIB plus an IORT boost. All of the surviving trial participants have achieved a minimum follow-up period of 60 months.

## 2. Methods

Retro-WTS was designed as a prospective single-center one-armed phase I/II study. The study design, as well as an unplanned interim analysis, have been published elsewhere [9,10]. In short, patients with histologically confirmed, primary or locally recurrent soft-tissue sarcoma of the retroperitoneal space judged to be at least marginally resectable were enrolled. Absence of primary metastases, tumor size of 5 cm or more were additional inclusion criteria. Exclusion criteria included desmoid tumors, gastrointestinal stroma tumors (GISTs), prior irradiation to the abdominal region, inflammatory bowel disease and incomplete staging. Immobilization was performed with individual body masks or vacuum mattresses. Planning was performed either with contrast-enhanced CT or MRI. Patients were treated with IMRT. The attempted dose was 45–50 Gy prescribed to the planning target volume (PTV) with a SIB of 50–56 Gy to the gross target volume (GTV) in 25 fractions. For target volume delineation, a 1.5 cm margin was added to the GTV to receive the CTV. CTV margins were reduced to respect the non-infiltrated adjacent organs at risk and anatomical borders. Surgery was scheduled approximately six weeks after the end of radiotherapy. Before surgery, re-evaluation with an abdominal CT or MRI was performed. An intraoperative radiation boost was dedicated to the whole tumor bed or the high-risk region for positive resection margins, which was defined by the surgeon together with the radiation oncologist. The patients received no pre- or postoperative chemotherapy. Regular follow-up visits including abdominal CT or MRI were performed every three months for the first two years, and every six months up to the end of the study follow-up interval of five years.

The primary objective of the trial was the LC rate after five years. The calculated sample size was 37 patients to detect an improvement in the 5-year LC rate from 50% to 70% with a statistical power of 80%. Data should be analyzed by the per protocol population and full-set population. Secondary endpoints included distant control (DC) and overall survival (OS). LC was defined as absence from abdominal recurrence. Data of those without recurrence were censored at the time of the last local MRI or CT. DC was defined as absence from distant metastases; data of those without distant progression were censored at the time of last thoracic CT. Timeframes were calculated from beginning of radiotherapy; survival analysis was performed by the Kaplan–Meier method. Data on acute toxicity and perioperative morbidity were published in an unplanned interim analysis [9].

The survival data of those lost to follow-up or those who were not followed with repetitive imaging after five years were updated by information from the German Cancer registry and the resident’s registration offices.

The study was approved by the Ethics Committee of Heidelberg University. Written informed consent was obtained from each patient prior to study entry.

## 3. Results

Between 2007 and 2017, a total of 37 patients with primary or recurrent (N = 6) retroperitoneal sarcomas were enrolled. The median age of the patients was 61.5 years (range 36–76 years); the gender distribution was homogeneous (male 49%, female 51%). The most common histology was dedifferentiated liposarcoma (51%), followed by leiomyosarcoma (24%) and well-differentiated liposarcoma (14%). The majority of lesions were high-grade (FNCLCC G1: 30%, G2: 38%, G3: 27%, two missing; Table 1); grading was determined at the time of the initial biopsy.

Of the 37 patients enrolled in the trial, 34 finished the neoadjuvant therapy per protocol. Percutaneous RT was performed as step-and-shoot IMRT in most cases (N = 32) and as helical IMRT in four cases. In total, four patients did not receive a SIB. The most common fractionation was 45 Gy in 25 fractions with a SIB up to 50 Gy (35%), followed by 45 Gy in 25 fractions with a SIB up to 54 Gy or 55 Gy (18% and 18%, Table 2). Gross total resection was performed in 35 cases. Two patients did not receive surgery; in one case, infiltration of the mesentery root was confirmed with intraoperative frozen sample analysis, and in the second case, inoperability was stated during surgery. On final pathology, the resection margin was mainly microscopic margin-positive R1 (N = 24, 69%). In one case, gross residual disease remained (R2, 3%). In two cases, the presence of residual tumor could not be assessed (RX, 6%), and in one case, the resection was described as marginal (3%). Seven patients received a microscopic margin-negative R0 resection (20%).

The IORT boost was performed in 75% of patients. The main reason for omission of IORT was the intraoperative difficulty in identifying a coverable high-risk region, as well as the fact that irradiating the whole tumor bed was not feasible due to its sheer size. One patient did not receive surgery due to the aforementioned infiltration of the mesentery root, but an IORT boost was applied. The most common IORT dose was 12 Gy (74%) prescribed to the 90% isodose; the most common energy applied was 8 MeV (60%).

The median follow-up of the survivors for OS was 109 months (range: 62–162 months). Of the 37 patients, 15 were alive and 22 deceased at the time of final analysis. Two patients died due to postoperative complications in the prolonged postoperative period, and one patient died 91 months after the beginning of RT and 10 months after his last follow-up presentation, at the age of 75, due to unknown reasons. The 5-year OS of the whole cohort was 59.5%. The 5-year OS of those treated per protocol accumulated to 68.8%.

Five patients were excluded from LC analysis per protocol. Of those, two patients had a preliminary termination of radiotherapy due to progression after 13 and 23 fractions, two patients did not receive surgery and one received upfront surgery without preoperative radiotherapy, as the tumor was rapidly progressing on planning CT.

**Figure 1:**
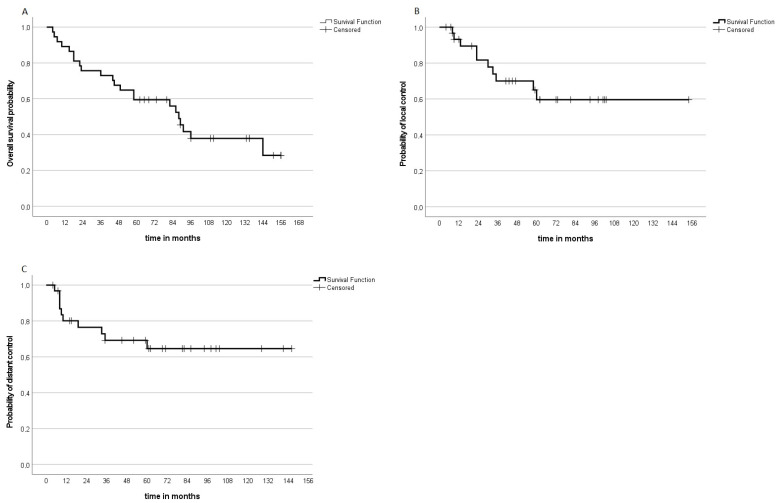
Kaplan–Meier analysis displays the probability of (**A**) overall survival, (**B**) local control and (**C**) distant control.

The median follow-up for LC of those without local progression was defined as the timeframe from the beginning of radiotherapy until the last abdominal MRI, or, in exceptional cases, CT. The median FU time for LC was 60.5 months (range: 4–154 months). In total, 10 patients had local progression during the observation interval, while 22 did not progress. Of ten patients, five progressed within two years and an additional five within five years. The 3-year LC was 70% and the 5-year LC was 59.6% (Figure 1). Of five patients treated per protocol for recurrent disease, three developed local progression, one developed distant progression and one developed local and distant progression.

Eleven patients received a total dose above 50.4 Gy plus IORT boost. The LC was 64.8%; however, the difference was not significant (*p* = 0.588). We did not identify factors influencing LC or OS on univariate analysis; only the comparison of recurrent vs. primary tumors showed a clear trend towards a higher LC probability for primary tumors (*p* = 0.006).

Of the 32 patients treated per protocol, 10 developed distant progression (4 pulmonary, 2 bone, 2 pulmonary and hepatic, 1 hepatic, 1 bone and soft tissue). Of eight leiomyosarcoma patients, five developed distant progression. The 5-year DC of those treated per protocol was 64.6%. On univariate analysis, the histology leiomyosarcoma was correlated with distant progression (*p* = 0.005).

## 4. Discussion

The role of preoperative radiotherapy in addition to surgery has been controversial for many years, and several contradicting retrospective studies have been published so far [11,12,13,14,15]. At present, only one prospective phase III trial for retroperitoneal sarcoma investigating this issue has completed patient recruitment. In the STRASS trial, 266 patients were enrolled and randomly assigned to surgery or preoperative radiotherapy of 50.4 Gy in 28 fractions followed by surgery. The results have been published preliminarily after a median follow-up of 43 months. The trial was powered for an increase of 20% in abdominal-recurrence-free survival (ARFS) at five years, which was not reached. The scientifically invalidated composite endpoint received much criticism after the full publication of the paper in Lancet Oncology in 2020. The data monitoring committee of the trial recommended performing additional analyses and modifying the endpoint. Thus, progression on preoperative imaging and becoming medically unfit where excluded from the primary endpoint in the second sensitivity analysis for those patients who had a subsequent macroscopic complete resection. It was demonstrated that the liposarcoma group had an increased 3-year ARFS of 75.5% after treatment with radiotherapy and surgery compared to 65.2% after surgery alone. As the trial was not powered to evaluate specific subtypes, the authors concluded that preoperative radiotherapy should not be considered the standard of care for retroperitoneal sarcomas [6]. The results of the STRASS trial were translated into quite contradictory clinical approaches. While some institutions decided not to offer preoperative radiotherapy to patients with retroperitoneal sarcomas outside of clinical trials, others have implemented a broader use of radiotherapy for patients with retroperitoneal well-differentiated liposarcoma. Valuable information on the topic was provided by the STREXIT results, published in 2022 by Callegaro et al. Additional 831 patients treated by the institutions participating in the STRASS trial were included in the analysis. A 1:1 propensity score matching was performed for 202 patients and the cohorts from STRASS and STREXIT were investigated in a pooled analysis. ARFS was defined as R2 resection, abdominal recurrence or death. Administration of radiotherapy was associated with an improved ARFS in patients with liposarcoma, especially in well-differentiated liposarcoma and G1–G2 dedifferentiated liposarcoma, while patients with leiomyosarcoma or G3 dedifferentiated liposarcoma did not benefit from radiotherapy [7]. The biological heterogeneity of retroperitoneal sarcomas and the different clinical behaviors clearly indicate a histology-tailored approach and management strategy. The next-generation STRASS 2 trial evaluates neoadjuvant chemotherapy in leiomyosarcoma and high-risk liposarcoma.

The results of the present trial did not reach the primary endpoint of an LC of 70% at five years. The 3-year ARFS in the aforementioned second sensitivity analysis of those who received preoperative radiotherapy and surgery in the STRASS trial was 71.3%. Our trial shows that relapse also occurs later than at three years; thus, an observational period of five years should be considered for further trials. The STRASS data will be published with a longer follow-up after five years, which will show whether the results achieved here are comparable to the results of bigger cohorts. The rate of microscopically complete R0 resections was 20%, but the role of R0 resection in RPS is controversial. Resection margins have been shown to be a strong prognostic factor for, at least, LC [4]. On the other hand, due to the retroperitoneal location with a close anatomical relationship to the spine and large blood vessels and the average tumor size of 15–30 cm, no large safety margins can be maintained during the resection. The evaluation of R1 resections in retroperitoneal sarcomas differs significantly from soft-tissue sarcomas of the extremities and trunk, and postoperative histopathological examination of the resection margins is less reliable [16]. In several publications, a distinction is only made between a macroscopically complete (R0/R1) and incomplete (R2) resection [3,6,17]. 

The survival rates at 3 years were lower, with 73% compared to the 84% of the STRASS trial [6], which might be also explained by the inclusion of recurrent tumors and the longer follow-up.

Prospective comparative data for the application of an IORT boost are, so far, not available. Nonetheless, IORT is considered to be well tolerated and a reasonable option to achieve dose escalation and improve LC, with a low risk of wound healing disorders or gastrointestinal toxicity [4,18,19,20]. Additional care should be taken to limit dose to the ureters and reduce the risk of ureter stenosis. The single prospective NCI trial identified neuropathy as a possible risk of IORT of the retroperitoneal space. Here, patients were randomized to postoperative high-dose RT (50 to 55 Gy) or IORT (20 Gy) in combination with postoperative percutaneous radiation therapy of 35 to 40 Gy [21].

Performing preoperative radiotherapy with a SIB above 50.4 Gy plus IORT boost was not possible or reasonable for several patients, but we observed increased rates of LC for those who received the combination of both. Applying a boost to the high-risk GTV (a smaller volume than the whole GTV, which constituted the SIB volume in this trial) is one additional option of dose escalation. The high-risk GTV generally includes the posterolateral abdominal wall, posterior retroperitoneal musculature, ipsilateral pre- and paravertebral space, major vessels or organs that will remain in situ after surgery [22]. This approach of neoadjuvant intensity-modulated proton therapy was investigated in a phase I pilot study (*n* = 11). The average-risk CTV received a dose of 50.4 Gy in 28 fractions and a SIB was performed to the high-risk CTV with 60.2 GyRBE, 61.6 GyRBE or 63 GyRBE. Beside one case of hydronephrosis, the treatment was tolerated well [8]. Further results of the phase 2 arm of the trial are eagerly awaited. In a plan comparison of 3D conformal proton therapy (3D CPT), intensity-modulated proton therapy (IMPT) and intensity-modulated photon therapy, 3D CPT and IMPT achieved lower organ-at-risk doses and IMPT achieved the closest conformity [23].

The standard neoadjuvant RT regimen is delivered in 25–28 fractions, but there is a growing interest in more condensed hypofractionated treatment approaches minimizing patient burden and psychological stress. Several ongoing trials are investigating different fractionation concepts for extremity and trunk soft-tissue sarcomas [24,25,26]. Particle therapy provides an improved dose distribution with a high dose conformity and reduction in dose to healthy tissue [27]. In analogy to our extremity soft-tissue sarcoma trial, we are currently investigating a hypofractionated particle treatment with carbon ions or protons with 13 fractions of 3 GyRBE single doses in a single-center, randomized, prospective phase II pilot trial for retroperitoneal sarcoma, combining the benefits of reduced organ-at-risk doses of protons or carbon ion with hypofractionation [28,29].

## 5. Conclusions

The data of a prospective phase II trial are presented. The strength of the cohort is the long follow-up in the prospective setting. The main limitation of the trial is the small sample size. The primary endpoint of a 5-year LC of 70% was not met. This might be explained by the inclusion of recurrent disease and the high rate of G3 lesions and leiomyosarcoma, which have been shown to profit less from radiotherapy in the time since the beginning of the trial. Stratification by grading and histology should be considered for future studies.

## Figures and Tables

**Table 1:** Patient characteristics.

		N	Range or Percent
		N = 37	
Age (median, range)		61	(36–76)
Gender			
	Male	18	49
	Female	19	51
Primary vs. recurrence			
	Primary	31	84
	Recurrent	6	16
Histology			
	Liposarcoma	26	70
	Leiomyosarcoma	9	24
	SFT	1	3
	NOS	1	3
Grading (FNCLCC)			
	G1	11	30
	G2	14	38
	G3	10	27
	Missing	2	5
Survival			
	Deceased	22	59
	Alive	15	41

**Table 2:** Treatment characteristics.

			N	Range or Percent
Neoadjuvant IMRT			N = 37	
	Completed		34	91
	Terminated prematurely		2	6
	Upfront surgery		1	3
Percutaneous RT technique			N = 36	
	Step-and-shoot IMRT		32	89
	Helical IMRT		4	11
Percutaneous RT				
Total dose for the main plan, boost and the number of fractions **			N = 34	
	SIB	Fx.		
45	50	25	12	35
45	54	25	6	18
50	55	25	6	18
41.4	46	23	1	3
45	50.4	25	1	3
45	55	25	1	3
50	-	25	3	9
50.4	-	28	2	6
55	-	25	1	3
45	-	25	1	3
Surgery			N = 37	
	Gross total		35	94
	Not performed		2	6
Resection margin			N = 35	
	R1		24	69
	R0		7	20
	RX		2	6
	R2		1	3
	Marginal		1	3
IORT			N = 36 *	
	Yes		27	75
	No		9	25
IORT total dose			N = 27	
	12 Gy		20	74
	15 Gy		4	15
	10 Gy		2	7
	20 Gy		1	4
IORT energy			N = 27	
	8 MeV		16	60
	6 MeV		9	33
	12 MeV		2	7
IORT cones (cm)				
Squircle (horseshoe-shaped)			N = 13	48
	6 × 7		2	
	7 × 8		2	
	10 × 10		1	
	10 × 11		2	
	10 × 13		4	
Straight round (diameter)			N = 5	19
	5		2	
	6		1	
	7		1	
	8		1	
Beveled round (diameter), angle up to 30%			N = 9	33
	5		1	
	6		2	
	7		1	
	8		4	
	9		1	

* one patient did not receive surgery after confirmation of infiltration of the mesentery root. He received IORT. ** preliminary cessation of RT excluded.

## Data Availability

The raw data supporting the conclusions of this article will be made available by the authors, without undue reservation.

## References

[1] Nussbaum D.P., Rushing C.N., Lane W.O., Cardona D.M., Kirsch D.G., Peterson B.L., Blazer D.G. (2016). Preoperative or postoperative radiotherapy versus surgery alone for retroperitoneal sarcoma: A case-control, propensity score-matched analysis of a nationwide clinical oncology database. Lancet Oncol..

[2] Raut C.P., Miceli R., Strauss D.C., Swallow C.J., Hohenberger P., van Coevorden F., Rutkowski P., Fiore M., Callegaro D., Casali P.G. (2016). External validation of a multi-institutional retroperitoneal sarcoma nomogram. Cancer.

[3] Gronchi A., Strauss D.C., Miceli R., Bonvalot S., Swallow C.J., Hohenberger P., Van Coevorden F., Rutkowski P., Callegaro D., Hayes A.J. (2016). Variability in Patterns of Recurrence After Resection of Primary Retroperitoneal Sarcoma (RPS): A Report on 1007 Patients From the Multi-institutional Collaborative RPS Working Group. Ann. Surg..

[4] Roeder F., Alldinger I., Uhl M., Saleh-Ebrahimi L., Schimmack S., Mechtersheimer G., Büchler M.W., Debus J., Krempien R., Ulrich A. (2018). Intraoperative Electron Radiation Therapy in Retroperitoneal Sarcoma. Int. J. Radiat. Oncol. Biol. Phys..

[5] Swallow C.J., Strauss D.C., Bonvalot S., Rutkowski P., Desai A., Gladdy R.A., Gonzalez R., Gyorki D.E., Fairweather M., van Houdt W.J. (2021). Management of Primary Retroperitoneal Sarcoma (RPS) in the Adult: An Updated Consensus Approach from the Transatlantic Australasian RPS Working Group. Ann. Surg. Oncol..

[6] Bonvalot S., Gronchi A., Le Péchoux C., Swallow C.J., Strauss D., Meeus P., van Coevorden F., Stoldt S., Stoeckle E., Rutkowski P. (2020). Preoperative radiotherapy plus surgery versus surgery alone for patients with primary retroperitoneal sarcoma (EORTC-62092: STRASS): A multicentre, open-label, randomised, phase 3 trial. Lancet Oncol..

[7] Callegaro D., Raut C.P., Ajayi T., Strauss D., Bonvalot S., Ng D., Stoeckle E., Fairweather M., Rutkowski P., van Houdt W.J. (2022). Preoperative Radiotherapy in Patients With Primary Retroperitoneal Sarcoma: EORTC-62092 Trial (STRASS) Versus Off-trial (STREXIT) Results. Ann. Surg..

[8] DeLaney T.F., Chen Y.-L., Baldini E.H., Wang D., Adams J., Hickey S.B., Yeap B.Y., Hahn S.M., De Amorim Bernstein K., Nielsen G.P. (2017). Phase 1 trial of preoperative image guided intensity modulated proton radiation therapy with simultaneously integrated boost to the high risk margin for retroperitoneal sarcomas. Adv. Radiat. Oncol..

[9] Roeder F., Ulrich A., Habl G., Uhl M., Saleh-Ebrahimi L., Huber P.E., Schulz-Ertner D., Nikoghosyan A.V., Alldinger I., Krempien R. (2014). Clinical phase I/II trial to investigate preoperative dose-escalated intensity-modulated radiation therapy (IMRT) and intraoperative radiation therapy (IORT) in patients with retroperitoneal soft tissue sarcoma: Interim analysis. BMC Cancer.

[10] Roeder F., Schulz-Ertner D., Nikoghosyan A.V., Huber P.E., Edler L., Habl G., Krempien R., Oertel S., Saleh-Ebrahimi L., Hensley F.W. (2012). A clinical phase I/II trial to investigate preoperative dose-escalated intensity-modulated radiation therapy (IMRT) and intraoperative radiation therapy (IORT) in patients with retroperitoneal soft tissue sarcoma. BMC Cancer.

[11] Bonvalot S., Rivoire M., Castaing M., Stoeckle E., Le Cesne A., Blay J.Y., Laplanche A. (2009). Primary retroperitoneal sarcomas: A multivariate analysis of surgical factors associated with local control. J. Clin. Oncol. Off. J. Am. Soc. Clin. Oncol..

[12] Le Péchoux C., Musat E., Baey C., Al Mokhles H., Terrier P., Domont J., Le Cesne A., Laplanche A., Bonvalot S. (2013). Should adjuvant radiotherapy be administered in addition to front-line aggressive surgery (FAS) in patients with primary retroperitoneal sarcoma?. Ann. Oncol. Off. J. Eur. Soc. Med. Oncol..

[13] Kelly K.J., Yoon S.S., Kuk D., Qin L.X., Dukleska K., Chang K.K., Chen Y.L., Delaney T.F., Brennan M.F., Singer S. (2015). Comparison of Perioperative Radiation Therapy and Surgery Versus Surgery Alone in 204 Patients With Primary Retroperitoneal Sarcoma: A Retrospective 2-Institution Study. Ann. Surg..

[14] Gronchi A., Lo Vullo S., Fiore M., Mussi C., Stacchiotti S., Collini P., Lozza L., Pennacchioli E., Mariani L., Casali P.G. (2009). Aggressive surgical policies in a retrospectively reviewed single-institution case series of retroperitoneal soft tissue sarcoma patients. J. Clin. Oncol. Off. J. Am. Soc. Clin. Oncol..

[15] Gronchi A., Miceli R., Colombo C., Stacchiotti S., Collini P., Mariani L., Sangalli C., Radaelli S., Sanfilippo R., Fiore M. (2012). Frontline extended surgery is associated with improved survival in retroperitoneal low- to intermediate-grade soft tissue sarcomas. Ann. Oncol. Off. J. Eur. Soc. Med. Oncol..

[16] Kirane A., Crago A.M. (2016). The importance of surgical margins in retroperitoneal sarcoma. J. Surg. Oncol..

[17] Toulmonde M., Le Cesne A., Mendiboure J., Blay J.Y., Piperno-Neumann S., Chevreau C., Delcambre C., Penel N., Terrier P., Ranchère-Vince D. (2014). Long-term recurrence of soft tissue sarcomas: Prognostic factors and implications for prolonged follow-up. Cancer.

[18] Petersen I.A., Haddock M.G., Donohue J.H., Nagorney D.M., Grill J.P., Sargent D.J., Gunderson L.L. (2002). Use of intraoperative electron beam radiotherapy in the management of retroperitoneal soft tissue sarcomas. Int. J. Radiat. Oncol. Biol. Phys..

[19] Krempien R., Roeder F., Oertel S., Weitz J., Hensley F.W., Timke C., Funk A., Lindel K., Harms W., Buchler M.W. (2006). Intraoperative electron-beam therapy for primary and recurrent retroperitoneal soft-tissue sarcoma. Int. J. Radiat. Oncol. Biol. Phys..

[20] Roeder F., Morillo V., Saleh-Ebrahimi L., Calvo F.A., Poortmans P., Ferrer Albiach C. (2020). Intraoperative radiation therapy (IORT) for soft tissue sarcoma—ESTRO IORT Task Force/ACROP recommendations. Radiother. Oncol..

[21] Sindelar W.F., Kinsella T.J., Chen P.W., DeLaney T.F., Tepper J.E., Rosenberg S.A., Glatstein E. (1993). Intraoperative Radiotherapy in Retroperitoneal Sarcomas: Final Results of a Prospective, Randomized, Clinical Trial. Arch. Surg..

[22] Baldini E.H., Bosch W., Kane J.M., Abrams R.A., Salerno K.E., Deville C., Raut C.P., Petersen I.A., Chen Y.L., Mullen J.T. (2015). Retroperitoneal sarcoma (RPS) high risk gross tumor volume boost (HR GTV boost) contour delineation agreement among NRG sarcoma radiation and surgical oncologists. Ann. Surg. Oncol..

[23] Chung C., Trofimov A., Adams J., Kung J., Kirsch D.G., Yoon S., Doppke K., Bortfeld T., Delaney T.F. (2022). Comparison of 3D Conformal Proton Therapy, Intensity-Modulated Proton Therapy, and Intensity-Modulated Photon Therapy for Retroperitoneal Sarcoma. Sarcoma.

[24] Koseła-Paterczyk H., Szacht M., Morysiński T., Ługowska I., Dziewirski W., Falkowski S., Zdzienicki M., Pieńkowski A., Szamotulska K., Świtaj T. (2014). Preoperative hypofractionated radiotherapy in the treatment of localized soft tissue sarcomas. Eur. J. Surg. Oncol..

[25] Pennington J.D., Eilber F.C., Eilber F.R., Singh A.S., Reed J.P., Chmielowski B., Eckardt J.J., Bukata S.V., Bernthal N.M., Federman N. (2018). Long-term Outcomes With Ifosfamide-based Hypofractionated Preoperative Chemoradiotherapy for Extremity Soft Tissue Sarcomas. Am. J. Clin. Oncol..

[26] Valle L.F., Bernthal N., Eilber F.C., Shabason J.E., Bedi M., Kalbasi A. (2021). Evaluating Thresholds to Adopt Hypofractionated Preoperative Radiotherapy as Standard of Care in Sarcoma. Sarcoma.

[27] Santos A., Penfold S., Gorayski P., Le H. (2022). The Role of Hypofractionation in Proton Therapy. Cancers.

[28] Brügemann D., Lehner B., Kieser M., Krisam J., Hommertgen A., Jaekel C., Harrabi S.B., Herfarth K., Mechtesheimer G., Sedlaczek O. (2022). Neoadjuvant irradiation of extremity soft tissue sarcoma with ions (Extrem-ion): Study protocol for a randomized phase II pilot trial. BMC Cancer.

[29] Seidensaal K., Kieser M., Hommertgen A., Jaekel C., Harrabi S.B., Herfarth K., Mechtesheimer G., Lehner B., Schneider M., Nienhueser H. (2021). Neoadjuvant irradiation of retroperitoneal soft tissue sarcoma with ions (Retro-Ion): Study protocol for a randomized phase II pilot trial. Trials.

